# Predictive and prognostic impact of preoperative complete blood count based systemic inflammatory markers in testicular cancer

**DOI:** 10.1590/S1677-5538.IBJU.2018.0820

**Published:** 2020-01-10

**Authors:** Ersan Arda, Gurkan Arikan, Hakan Akdere, Murat Akgul, Ilkan Yuksel

**Affiliations:** 1 Department of Urology Trakya University School of Medicine Edirne Turkey Department of Urology, Trakya University School of Medicine, Edirne, Turkey;; 2 Department of Urology Namik Kemal University School of Medicine Tekirdağ Turkey Department of Urology, Namik Kemal University School of Medicine, Tekirdağ, Turkey

**Keywords:** Testicular Neoplasms, Neutrophils, Lymphocytes

## Abstract

**Purpose:**

To determine the utility of preoperative complete blood count (CBC) based systemic inflammatory markers in the prediction of testicular cancer and its prognosis.

**Material and Methods:**

Between 2008-2017 the data of all testicular tumor patients undergoing radical orchiectomy were retrospectively analyzed. Patient baseseline characteristics (age, tumor stage, tumor markers, etc.) and results of routine preoperative blood tests including mean platelet volume (MPV), red cell distribution width (RDW), lymphocyte ratio (LR) and neutrophil ratio (NR) were retrieved. In addition, neutrophil to lymphocyte ratio (NLR) was calculated.

**Results:**

Mean age of the tumor and control group was 36.0±15 and 30.50±11 years, respectively. Mean RDW, NR and NLR were significantly higher in the tumor group with p values<0.001; whereas LR and MPV were significantly higher in the control group (p<0.001). Receiver Operating Characteristic (ROC) analyses of LR, NR, RDW, MPV, and NLR are shown in Table-3. The cut off values for RDW and NR were found as 13,7 (Area under the curve (AUC): 0.687, sensitivity = 42.2%, specificity = 84.8%) and 55.3 (AUC:0.693, sensitivity 72.2%, specificity 62%), respectively. Area under the curve for NLR in tumor group was 0.711, with a threshold value of 1.78 and sensitivity=81.8% and specificity=55.4% (AUC:0.711/sig<0.001) that together with RDW exhibited the best differential diagnosis potential which could be used as an adjuvant tool in the prediction of testicular tumor and its prognosis.

**Conclusion:**

Several systemic inflammatory markers, which are obtained by routinely performed cost-effective blood tests, could demonstrate incremental predictive and prognostic information adjuvant to preoperativly achieved testiscular tumor markers.

## INTRODUCTION

Testicular cancer is the most common malignancy in men between the ages of 20-40 and constitutes about 1-1.5% of all cancers. Mainly, it can be classified as germ cell, sex cord-gonadal stromal and secondary testicular tumors (1). Clinical admission, in daily urology practice, is usually due to painless testicular mass, while serum tumor markers (human chorionic gonadotropin (HCG), alpha-fetoprotein (AFP) and lactate dehydrogenase (LDH) and scrotal ultrasonography are used for definitive diagnosis (2).

Studies investigating the relationship of inflammation and cancer, by revealing different mechanisms of influence, are increasing in recent years. It has been reported that tumor cells are triggering cancer-related inflammation, and increasing tumor promoting effect, by secreting chemokines, cytokines and prostaglandins (3). Particularly, in various urinary tract malignancies such as renal cell carcinoma (RCC), bladder cancer and prostate cancer, it has been claimed that cancer-related inflammation plays an important role in disease progression and prognosis (4). In addition, a recent study showed that chronic inflammatory processes such as orchitis-epididymorchitis can influence the formation of testicular malignancy (5). Therefore, taken into account the role of inflammation in tumor biology, using inflammatory markers such as neutrophil ratio (NR), lymphocyte ratio (LR) and/or neutrophil to lymphocyte ratio (NLR) in predicting the occurence and prognosis of the disease, is of great importance (6). Among these markers, it has been suggested that, especially NLR reflects immune response and can be used as a prognostic marker, in many urologic malignancies (7). Nowadays, as a result of previously accomplished studies, the European Urological Association approved NLR as a prognostic parameter for RCC (8).

Even though inflammatory processes comprise a more important role in testicular tissue than other urinary tract organs, there are only a few studies showing the relationship between inflammatory markers and testicular cancer.

Based on the above mentioned hypothesis, we aimed to determine the utility of preoperative complete blood count (CBC) based systemic inflammatory markers in the prediction of testicular cancer and its prognosis.

## MATERIAL AND METHODS

Between 2008-2017, the data of all testicular tumor patients, who underwent radical orchiectomy in two University Hospitals in the Region of Trakya (Turkey) were retrospectively examined. Histopathologically proven testicular germ cell tumor patients of stage pT1-T4 including any regional lymph node positivity without distant organ metastasis, and healthy unoperated grade 1 varicocele individuals with peripheral blood count results, were included in the study and defined as tumor and control group, respectively.

Patient baseline characteristics (age, tumor stage, etc.) and routine preoperative blood test results were retrieved from both hospitals electronic data base. Biochemical analyses included testicular tumor markers (AFP, HCG, LDH) and CBC-based systemic inflammatory markers such as mean platelet volume (MPV), red cell distribution width (RDW), LR and NR. Additionally to the extracted CBC based markers, NLR was calculated to compare between the groups.

The following exclusion criterias for both groups were used: i) men with a secondary malignancy: ii) presence of another active infection: iii) disease causing increased inflammatory response (e.g. familial mediterranean fever): iv) hematologic diseases affecting blood count: and/or v) receiving chemotherapy, meanwhile.

Definition of cancer specific survival (CSS) was the time period (in months) involving time of surgery to cancer related death. Postoperative follow-up of testicular tumor patients in both clinics were routinely performed according to the EAU guideline protocol (9). Informed consents were taken and institutional review board was approved by the hospital ethics committee.

### Statistical analysis

Statistical analyses were performed by R version 3.5.3 (2019-03-11). Continuous variables were expressed as means (standard deviation (SD)) or median (interquartile range (IQR)) where appropriate, categorical variables were expressed as frequencies and percentages. Variables were compared for statistical significance between groups by Student t-test or Mann Whitney U test. The assocaiton between categorical variables was tested using Fisher’s exact test. Kaplan Meier analysis was used to correlate NLR with CSS. Receiver operating characteristic (ROC) curve analyses were performed to assess the discriminative ability of the biomarkers for testicular cancer. The cut-off points for biomarkers were determined by a criterion based on Youden’s Index defined as Y I(c)=maxc(Se(c)+Sp(c)−1) and the corresponding specificity-sensitivity levels were provided.

For all analyses, the p value of p <0.05 was considered statistically significant.

## RESULTS

After determining inclusion/exclusion criterias, a total of 182 patients were included in the study and divided into two groups, as tumor and control groups, with 90 and 92 patients each, respectively. Descriptive properties and their distribution with respect to groups are summarized in Table-1.

**Table 1:** Descriptive statistics and comparisons of CBC based parameters with respect to groups.

Variables	Tumor group (n=90)	Control group (n=92)	P value
**Age** (years)	36 (15) min:20; max:76	30.50 (11) min:16; max:54	**<0.001**
**LR** (%)	26.15 (14.23) min:5.5; max:80	33.90 (11.78) min:11.10; max:13.30	**<0.001**
**NR** (%)	63.20 (13.10) min:6; max:87.2	53.95 (13.65) min:33.6; max:81.9	**<0.001**
**NLR** (%)	2.37 (2.02) min:0.33; max:15.85	1.6 (1.05) min:0.58; max:7.38	**<0.001**
**MPV** (10^3^/uL)*	8.35 (1.17) min:6.02; max:11.4	9.09 (2.33) min:6.10; max:13.3	**<0.001**
**RDW** (10^3^/uL)	13.50 *(*1.47) min:11.50; max:25.80	12.9 (0.85) min:11.6; max:17	**<0.001**

**LR =** Lymphocyte ratio; **MPV =** Mean platelet volume; **CBC =** Complete blood count; **NLR =** Neutrophil/Lymphocyte ratio; **RDW =** Red cell distribution width; **NR =** Neutrophil ratio

*: The variables with asterisk are described by mean (standard deviation), and the corresponding p value is based on t-test, otherwise the variables are described by median (interquartile range) and the corresponding p value is based on Mann Whitney U test.

Mean age of the tumor and control groups was 36.0±15 and 30.50±11 years, respectively. Pathological subtypes consisted of 90 germ cell tumors including 42 seminomas (46.6%), 48 non-seminoma (53.4%), with a mean follow-up period of 39.83 months. According to the TNM classification, patients comprised of 56 (62.2%) pT1, 28 (31.1%) pT2, 4 (4.4%) pT3 and 2 (2.2%) pT4 patients (Table-2). Mean RDW, NR and NLR were significantly higher in the tumor group with p values <0.001, whereas LR and MPV were significantly higher in the control group (p <0.001). During follow-up, postoperative 68 patients of the tumor group got an additional treatment such as chemotherapy (n=62), radiotherapy (n=3) or chemoradiotherapy (n=3).

**Table 2:** Tumor Classification and Pathological Stage

Subtypes of Tumor		Number (n)	Percentage (%)
**GERM CELL TUMORS (n=90)**	**Seminoma**		
	pT1	26	28.9
	pT2	15	16.7
	pT3	-	-
	pT4	1	1.1
	**Mix germ cell**		
	pT1	25	27.8
	pT2	13	14.4
	pT3	3	3.3
	pT4	1	1.1
	**Yolc sac**		
	pT1	2	2.2
	**Immature teratoma**		
	pT1	2	2.2
	**Mature teratoma**		
	pT3	1	1.1
	**Germ cell neoplasy**		
	pT1	1	1.1

**Figure 1:**
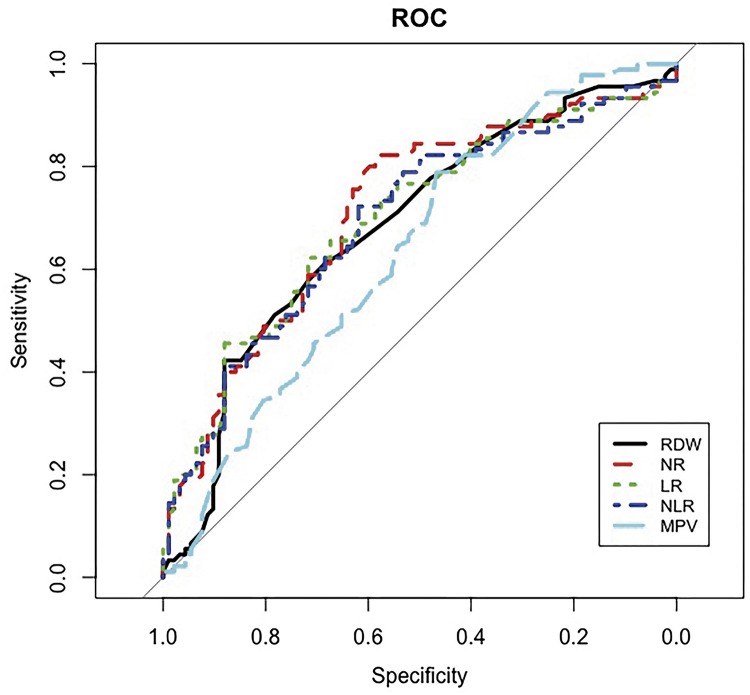
Optimal cut-off values and ROC analyses for LR, NR, RDW, MPV and NLR.

Receiver Operating Characteristic (ROC) analyses of LR, NR, RDW, MPV and NLR are shown in Table-3 and Figure-1. The cut off values for RDW and NR were found as 13.7 (Area under the curve (AUC): 0.687, sensitivity = 42.2%, specificity = 84.8%) and 55.3 (AUC: 0.693, sensitivity = 72.2%, specificity = 62%), respectively. Area under the curve for NLR in tumor group was 0.711, with a threshold value of 1.78 and sensitivity=81.8% and specificity=55.4% (AUC: 0.711/sig <0.001) that together with RDW exhibited the best differential diagnosis potential which could be used as an adjuvant tool in the prediction of testicular tumor and its prognosis (Table-3).

**Table 3:** Optimal cut-off values and ROC analyses for LR, NR, RDW, MPV and NLR.

Variables	AUC	Cut-off	Sensitivity	Specificity	PPV	NPV
**Preop RDW**	0.687	>13.7	42.2%	84.8%	73.1%	60%
**Preop NLR**	0.711	>1.78	81.8%	55.4%	64.3%	76.1%
**Preop NR**	0.693	>55.3	72.2%	62%	65%	70%
**Preop LR**	0.698	<28.7	62.2%	70%	67%	65%
**Preop MPV**	0.636	<33.4	78.9%	44.6%	58.2%	68.3%

**RDW =** Red cell distribution width; **NR =** Neutrophil ratio; **NLR =** Neutrophil to Lymphocyte ratio; **MPV =** Mean platelet volume; **LR =** Lymphocyte ratio; **AUC =** Area under the curve; **PPV =** Positive Predictive Value; **NPV =** Negative Predictive Value; >: greater than, <: smaller than

Distribution of descriptive properties and comparison of clinical parameters between the patients with respect to NLR cut-off values (<1.78 and ≥1.78) are shown in Table-4. Except HCG, NR, LR and NLR no statistically significant difference was found between both groups.

**Table 4:** Distribution of descriptive properties and comparison of clinical parameters between the patients with respect to NLR of <1.78 and ≥1.78.

Variables	NLR <1.78 (n=25)	NLR ≥1.78 (n=65)	P value
**Age (years), Median (IQR)**	36.00 (12.00)	36.00 (16.00)	0.576
**Tumor size (cm**^3^**), Median (IQR)**	6.00 (45.40)	13.50 (67.00)	0.313
**Follow-up (months), Median (IQR)**	36.00 (36.00)	24.00 (48.00)	0.282
**Stage, n(%)**			0.475
T1	17 (68.00%)	38 (58.46%)	
n>T2	8 (32.00%)	27 (41.54%)	
**Tumor markers, Median (IQR)**			
Preop AFP (U/mL)	4.50 (10.28)	5.10 (147.14)	0.385
Preop HCG (mIU/mL)	1.00 (4.65)	5.00 (41.00)	0.047*
Preop LDH (U/L)	310.00 (242.00)	335.00 (386.00)	0.859
**CBC based markers, Median (IQR)**			
Preop NR (%)	52.00 (11.00)	68.10 (11.00)	<0.001*
Preop LR (%)	36.30 (6.70)	22.10 (10.60)	<0.001*
Preop NLR (%)	1.38 (0.42)	3.12 (2.27)	<0.001*
Preop MPW (10^3^/uL)	8.00 (1.90)	8.20 (1.60)	0.491
Preop RDW (10^3^/uL)	13.30 (2.10)	13.50 (1.20)	0.695
**Lymph node metastasis at the time of diagnosis, n (%)**	2 (8.00%) 23 (92%)	9 (13.85%) 56(86.15%)	0.721
Prognostic factors related to occult metastatic disease*, n (%)	8 (32.00%) / +17 (62.00%) / -	27 (41.54%) / + 38 (58,36%) / -	0.475
**CSS, n (%)**	19 (76.00%)	48 (73.38%)	1
Survived Ex	6 (24.00%)	17 (26.62%)	

**AFP =** Alfa feto protein; **HCG =** Human chorionic gonodotropin; **LDH =** Lactate dehidrogenase; **LR =** Lymphocyte ratio; **NR =** Neutrophil ratio; **MPW =** Mean platelet volume; **NLR =** Neutrophil/Lymphocyte ratio; **RDW =** Red cell distribution width.

* Rete testis invasion, Lymphovascular invasion

Mean NLR of 42 patients with stage pT1, and 48 patients with stage ≥pT2, was 3.06 and 3,15, respectively. Neutrophil to lymphocyte ratio was quantitatively higher in patients with a stage ≥T2, however no statistically significant difference between both groups was observed (p=0.107). Retroperitoneal lymph node metastases, at the time of disease diagnosis, was revelead in two of 25 patients (8.00%) with a NLR <1.78 and in nine of 65 patients (13.85%) with a NLR ≥1.78.

**Figure 2:**
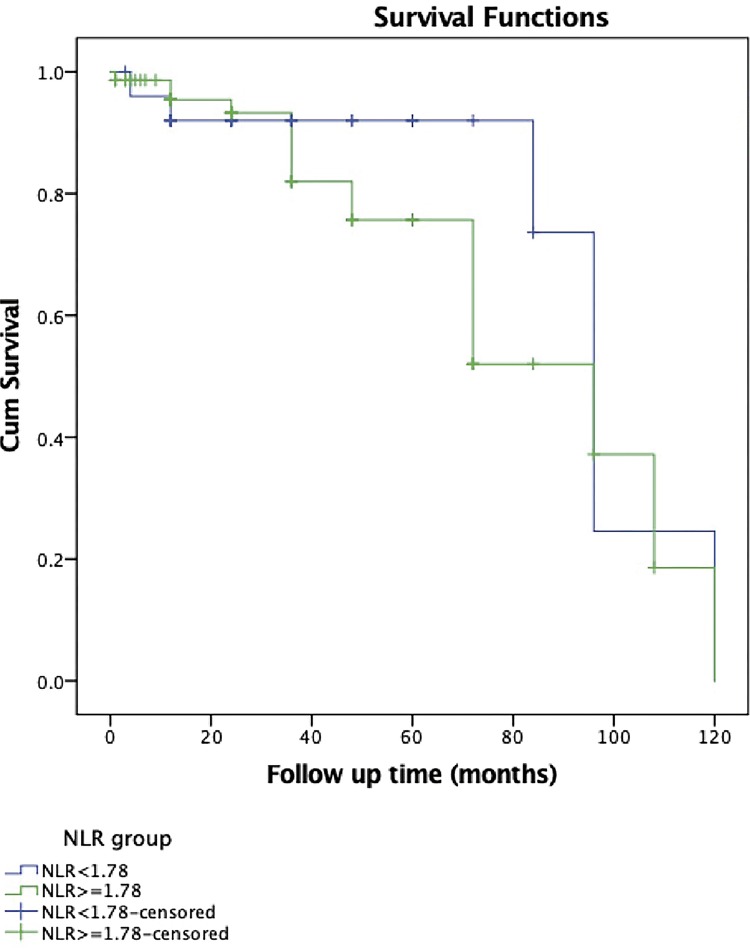
Kaplan-Meier curves used to evaluate the correlation between neutrophil-to lymphocyte ratio and CSS.

Mean CSS of all patients was calculated as 84.78±4.89 months, whereas median CSS was found 96 month showing no difference between the groups according to NLR cut-off value (p=0.378) (Figure-2) In addition, five year overall survival rates for patients with NLR <1.78 and ≥1.78 were found as 54.00% and 48.00%, respectively.

## DISCUSSION

Immune mechanisms, such as the role of neutrophils as a promoter in tumor formation and the presence of antitumoral activity of lymphocytes have been reported to be associated with malignancies (10). Studies have shown that systemic inflammatory markers, including cytokines, C-reactive protein (CRP), albumin, serum amyloid A and leukocytes, may be independent prognostic factors in cancer patients (11). Recently, in addition to those markers, NLR and platelet lymphocyte ratio (PLR) have been extensively investigated and their relation to the formation and progression of malignancy were reported (10). Especially in urinary system malignancies like prostate, bladder and kidney cancers, the role and efficiency of NLR has been shown in several studies (6, 12-14).

Researchers investigating the relationship between tumor size and NLR in patients with RCC, demonstrated that NLR was significantly higher in patients with a tumor size larger than 4cm (15). Considering that RCC’s larger than 4cm are upstaged according to the TNM classification, it was thought that NLR may correlate with tumor stage. Based on this suggestion, the obtained results of our testicular tumor patients revealed a significant correlation between tumor stage and the NLR cut off value, supporting previously accomplished studies (15, 16). Apart from this, a study evaluating ovarian mature cystic teratoma patients, it was reported that NLR was significantly higher in patients that showed malignant transformation (7). Interpreting our results, besides the similarity of embriological origin and exposion of frequent inflammatory processes identical to the testes, this finding could contribute to the literature in terms of showing the association of NLR and genitourinary related malignancies.

We sought to describe the potential association between preoperative CBC-based blood count parameters and testicular cancer patients who underwent radical orchiectomy. The major findings of the present study are that: i) RDW, NR and NLR are significantly higher in testicular tumor patients compared to healthy control subjects, and ii) especially NLR and RDW could be used as a predictive and/or prognostic factor showing the highest sensitivity and specificity, respectively.

In a study comparing 36 patients with localized testicular cancer and 36 control subjects, it was shown that NR and NLR were significantly higher in the tumor group, whereas LR was higher in the control group, which was in agreement to our findings (4). However, ROC analysis revealed a significantly lower NLR cut off value and sensitivity compared to our results, which we believe to be caused due to the small sample size of this study.

Jankovich et al. (17), studied 103 testicular germ cell tumor (GCT) patients and evaluated the prevalence of their histopathology, metastatic status and tumor stage according to NLR <4 or NLR ≥4. Despite that, NLR cut off value was considerably higher than ours, no statistically significant difference according to these parameters was observed, which was supported by our findings.

In another study conducted by Bolat et al., patients were divided and compared according to the NLR treshold value of <3.55 and ≥3.55, to investigate its potential impact on the prognosis of testicular GCT. Similar to our findings, no significant relation between NLR and tumor stage or prognostic factors related to occult metastasis, like rete testis invasion and/or lymphovascular invasion, was obtained. However, the average sample size and inclusion of distant organ metastatic patients to such a small group, made the clinical evaluation of their data difficult and query (18).

Despite the adequate patient size, the retrospective nature and the inability of comparing extently matched metastatic and non-metastatic patients, according to NLR and other inflammatory markers, are considered the main limitations of the present study. Additionally, due to treatment strategies of testicular masses we could not consider the circadien rhythm of neutrophils and lymphocytes during blood sample collection. Apart from these, it could be interesting to evaluate preoperative to postoperative alterations of NLR and other CBC based inflammatory parameters after tumor removal and to compare the sensitivity and specificificity against established routine testis tumor markers.

## CONCLUSIONS

Several systemic inflammatory markers, which are obtained by routinely performed cost-effective blood tests, could demonstrate incremental predictive and prognostic information adjuvant to preoperativly achieved testicular tumor markers. However, further research is needed.
